# Environmental Fate of Chiral Herbicide Fenoxaprop-ethyl in Water-Sediment Microcosms

**DOI:** 10.1038/srep26797

**Published:** 2016-05-26

**Authors:** Xu Jing, Guojun Yao, Donghui Liu, Mingke Liu, Peng Wang, Zhiqiang Zhou

**Affiliations:** 1 Beijing Advanced Innovation Center for Food Nutrition and Human Health. Department of Applied Chemistry, China Agricultural University, Beijing, 100193, P. R. China

## Abstract

The environmental fate of the herbicide fenoxaprop-ethyl (FE) in water, sediment and water-sediment microcosm was studied and degradation products fenoxaprop (FA), ethyl-2-(4-hydroxyphenoxy)propanoate (EHPP), 2-(4-hydroxyphenoxy)propanoic acid (HPPA) and 6-chloro-2,3-dihydrobenzoxazol-2-one (CDHB) were monitored. FE, FA, EHPP and HPPA were chiral and the environmental behavior was investigated on an enantiomeric level. In water, sediment and water-sediment microcosms, fenoxaprop-ethyl degraded very fast with half-lives less than 1 day and it was found the herbicidally inactive S-enantiomer degraded faster. Fenoxaprop was the main primary degradation product which was quickly formed and the further degradation was relatively slow with half-lives of 6.4–12.4 days, and the S-enantiomer degraded faster too. EHPP, HPPA and CDHB could be found and S-EHPP and S-HPPA were degraded preferentially. The effects of microorganism and water content were investigated and it was found that the enantioselectivity was attributed to microorganisms. In sediment, the main degradation pathway of fenoxaprop-ethyl was hydrolysis and the degradation rate of fenoxaprop-ethyl increased with water content. The degradation products and enantioselectivity should be considered for the impact of fenoxaprop-ethyl on the aquatic system.

Fenoxaprop-ethyl (FE, Fig. 1), (±)-ethyl 2-[4-[(6-chloro-2-benzoxazolyl)oxy]phenoxy]propanoate, a selective aryloxyphenoxypropionate herbicide, is registered for postemergence control of various annual and perennial grass weeds in dicotyledonous crops as well as grains by inhibiting fatty acid synthesis through inhibiting acetyl-CoA carboxylase^1^,^2^. Since 1990s, FE has been widely applied to selectively control weeds in soybean, sugar beet, cotton, potato and wheat fields^3^.

Incidentally, pesticide applied to agricultural fields may reach various water bodies via spray-drift and runoff events and successively undergo degradation processes such as hydrolysis, photolysis, and microbial transformations together with partitioning to suspended matters and bottom sediment. The degradation of FE has been studied and its main degradation pathway was found to be hydrolysis^4^,^5^. Because of the breakdown of the ester bond of FE, the parent compound is hydrolyzed to its corresponding acid fenoxaprop (FA, Fig. 1). The general properties of FE and FA listed in the Table S1. The degradation products EHPP, HPPA and CDHB (Fig. 1) derive from the breakdown of the benzoxazolyl-oxyphenyl ether linkage. According to the literatures, FA formed by hydrolysis, while CDHB and EHPP may form by photolysis or hydrolysis^6^,^7^. FE has high toxicity to aquatic organisms^8^: LC_50_ (96 h) for rainbow trout is 0.57 mg/L; EC_50_ (48 h) for *Daphnia magna* is 0.56 mg/L; LC_50_ (72 h) for *Scenedesmus subspicatus* is 0.51 mg/L. The degradation products may be more toxic and persistent^6^,^7^. For instance, the degradation product CDHB may cause toxic effects on the growth of water invertebrates, bacteria, fungi, even plants^6^. Products 4-[(6-chloro-2-benzoxazolyl)oxy]phenol and hydroquinone were more toxic to *Daphnia magna* than the parent FE^7^. However, little information has been reported about the fate of FE especially in the complex environmental systems, and there is a need to consider the degradation products.

About 25% of the pesticides sold were chiral in 1996. Nowadays, it is estimated that chiral pesticides would account for more than 40% of the currently used pesticides in China^9^. Environmental behavior of the two enantiomers may be totally different. Fenoxaprop-ethyl is a chiral herbicide because there is a chiral carbon center in the chemical structure, and thus has two enantiomers. But the herbicide activity mostly originates from the R-enantiomer^10^,^11^. The main primary degradation product FA is the active component for weed control which is also chiral with the R-enantiomer more active than the S-enantiomer^12^. The degradation and metabolism of FE and its chiral degradation product FA have been found to be enantioselective^10^,^11^,^13^. In three soils, S-FE and S-FA degraded faster than the R-enantiomer^10^. The metabolism of FE in rabbits *in vitro* demonstrated that S-FE preferentially degraded in plasma. FE degraded very fast to the metabolite FA in rabbits *in vivo*, and S-FA degraded faster than R-FA in plasma, heart, lung, liver, kidney and bile^11^. Enantioselective environmental behaviors of chiral pesticides and their chiral degradation products should be taken into account for an accurate risk assessment and correct use of chiral pesticides^14^.

This work was designed to study the environmental behavior of FE in aquatic system using water–sediment microcosms. The different degradations of the enantiomers of FE and the chiral degradation products FA, EHPP and HPPA were also investigated. The effects of microorganisms and water content on the degradation were studied. To our knowledge, it has not been reported the enantioselective degradation of FE, FA, EHPP and HPPA in water, sediment and water-sediment microcosm and few information has been known about the environmental behavior of the degradation products EHPP, HPPA and CDHB. The work may supply some information to evaluate the impacts of FE on the aquatic system.

## Results and Discussion

### Degradation of FE in water and sediment

**Figure 2:**
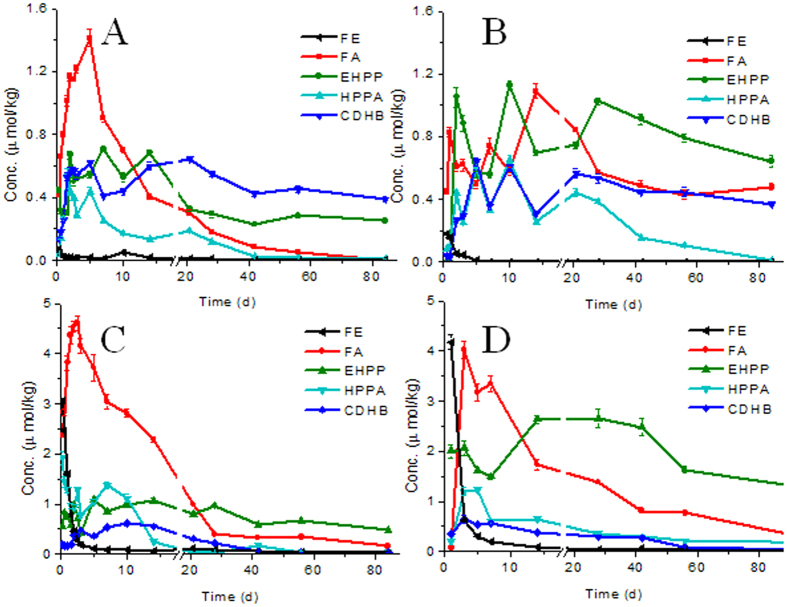
Concentration-time curves of FE and its degradation products in (**A**) water; (**B**) sterilized water; (**C**) sediment; (**D**) sterilized sediment. Starting concentration of FE in water, sterilized water, sediment and sterilized sediment were 1.38, 1.38, 6.91 and 6.91 μmol/kg, respectively.

**Figure 3:**
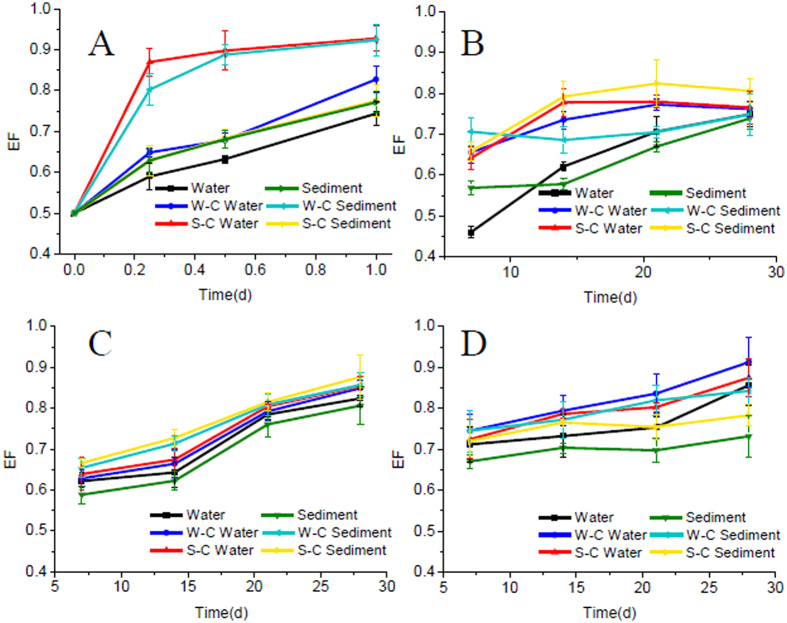
EF values-time curves of FE and its degradation products in water, water of W-C microcosm, water of S-C microcosm, sediment, sediment of W-C microcosm, sediment of S-C microcosm. (**A**) FE; (**B**) FA; (**C**) EHPP; (**D**) HPPA.

FE decreased rapidly with time in water (Fig. 2A) and sediment (Fig. 2C) from reservoir, with more than 98% degraded after 2 days. FE degraded more rapidly in water than in sediment. The EF values, defined as the concentration ratio of R-enantiomer to the sum of S- and R-enantiomer, were greater than 0.500, which meant that R-enantiomer had higher residue concentrations than S-enantiomer. A preferential degradation of the S-enantiomer of FE was found in both water and sediment (Fig. 3) from reservoir. Half-lives (t1/2) were calculated according to the first-order kinetics analysis model. The half-lives of both enantiomer of FE in water were below 0.25 d, and the half-lives of S-FE and R-FE in the sediment were 0.4 and 0.7 d, respectively (Table 1). The EF values increased from the initial value of 0.491 (0 d) to 0.744 in water and 0.771 in sediment after 1 day (Fig. 3A).

The concentration of the primary degradation product FA from the breakdown of the ester bond increased to a maximum value at day 5 in the water (Fig. 2A) and at day 2.5 in the sediment (Fig. 2C) respectively and then decreased. In the early stage of the degradation, S-FA was preferentially formed in the sediment, and then degraded faster than R-enantiomer. The half-lives of S-FA and R-FA in water were 8.2 and 12.4 d, respectively (Table 1). EF values were below 0.500 in the first seven days, and then increased gradually (Fig. 3B). Similar results were found in the sediment with half-lives of S-FA and R-FA of 6.4 and 9.4 d, respectively (Table 1), indicating a faster degradation in sediment than in water. EF values changed from 0.410 (6 h) to 0.749 (28 d) in the sediment (Fig. 3B).

The degradation product EHPP degraded faster in water than in sediment, in contrast, HPPA degraded slower in water than in sediment (Table 1). S-EHPP and S-HPPA eliminated preferentially in both water and sediment (Fig. 3C,D). CDHB was persistent with half-life of 96.3 d in water and 17.8 d in sediment.

Half-lives (Table 1) were in the order of FE < FA < HPPA < EHPP < CDHB in water, and FE < HPPA < FA < CDHB < EHPP in sediment. At day 84, the main residues in water were EHPP and CDHB and in sediment were FA and EHPP (Fig. S1).

### Degradation and distribution of FE in water-sediment microcosms

The degradation behavior of the enantiomers was investigated in water-sediment microcosms and two contamination ways were compared: water contamination (FE was fortified in water from reservoir) and sediment contamination (FE was fortified in sediment from reservoir). The concentration variations of FE and its degradation products in the water contamination (W-C) microcosm and sediment contamination (S-C) microcosm with time were shown in Fig. S2.

FE degraded in the order: sediment (T_1/2_, 13.1 h) < S-C (T_1/2_, 11.9 h) < W-C (T_1/2_, <6 h) < water (T_1/2_, <6 h). Significant degradation differences existed between W-C and S-C microcosms because FE degraded much more rapidly in water than in sediment. In order to compare the overall formation and dissipation of a degradation product in water, sediment and the water-sediment microcosms, the concentration-time curves were generated by the sum of a degradation product in the system with time (Fig. S3). For the degradation products, there were minor discrepancies between the two contamination ways maybe because there was enough time to distribute in the two phases. At day 84, the main residue was EHPP in the two microcosms (Fig. S1).

**Figure 4:**
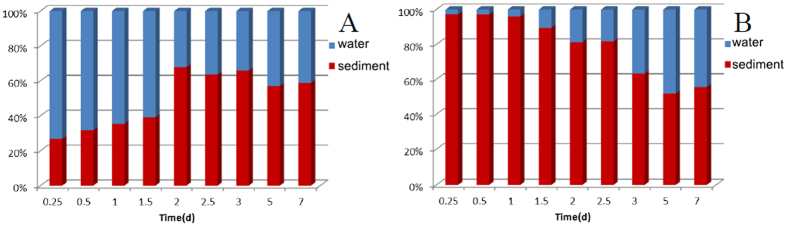
Distribution of FE in water and sediment in (**A**) W-C microcosm; (**B**) S-C microcosm.

The distribution of FE between water and sediment in the water-sediment microcosms with time was investigated. The amount in the sediment gradually tended to reach phase equilibrium and occupy about 60% after 3 days no matter by water or sediment contamination (Fig. 4). At the first time point (6 h) of the water contaminated (W-C) microcosm, more than 20% of FE was detected in the uncontaminated sediment (Fig. 4A), while FE in the sediment contaminated (S-C) microcosm barely transferred from the contaminated bottom sediment to the uncontaminated water because of its poor aqueous solubility (Fig. 4B). Fast distribution of the degradation products between water and sediment was also found. More than 20% of each of the degradation products was found in the uncontaminated water and sediment at the first time point (6 h). Bioturbation of benthonic organism under real environmental conditions may accelerate the distribution between water and sediment.

The chiral profiles of FE, FA, EHPP and HPPA in the two water-sediment microcosms were shown in Fig. 3A–D indicating the S-enantiomers of FE, FA, EHPP and HPPA being degraded faster in both water and sediment in the two microcosms. In general, EF values in the water-sediment microcosms were higher than those in water or sediment. Both overlying water and bottom sediment attributed to the enantioselectivity.

### Effect of microorganisms

Sterilization treatments of water and sediment from reservoir were performed to determine the impact of microorganisms on the degradation and enantioselectivity. The degradation of FE and its degradation products turned to be slower under sterilized conditions (Fig. 2 and Table 1). For example, the half-lives of FE in sterilized water and sediment were 1.3 and 1.4 days, but it was below 0.25 and 0.8 day in unsterilized water and sediment. The amount of FA produced under sterilized conditions was less, with maximum value of 1.09 μmol/kg in sterilized water and 4.02 μmol/kg in sterilized sediment (Fig. 2). However, the amount of EHPP produced under sterilized conditions was approximately twice about that under unsterilized conditions, indicating faster conversion of FE to EHPP under sterilized conditions. The maximum value of EHPP was 1.13 and 2.65 μmol/kg in sterilized water and sediment. A little more HPPA was also observed under sterilized conditions which were from the degradation of EHPP. There were no obvious differences in the formation of CDHB between sterilized and unsterilized conditions. Microbial degradation is the main pathway for many pollutions. In this study, mass balance was not changed much in the degradation process of FE to FA. The process was largely driven by hydrolysis and less affected by microorganisms. The total mass gradually decreased with the dissipation of the degradation products. From the result of the experiment, microorganisms might play an important role in the dissipation of the degradation products and thus cause the total mass loss.

According to the previous research, the enantioselectivity of FE and FA in soils was attributed to microbially mediated processes^10^. Sterilization treatment was a simple way to determine the impact of microbial process on the enantioselective degradation. EF values remained unchanged from the initial value during the incubation period in the sterilized water and sediment indicating no enantioselective degradation happened. The results confirmed the enantioselectivity of FE, FA, EHPP and HPPA in water and sediment was controlled by a biotic pathway.

### Effect of water content

**Figure 5:**
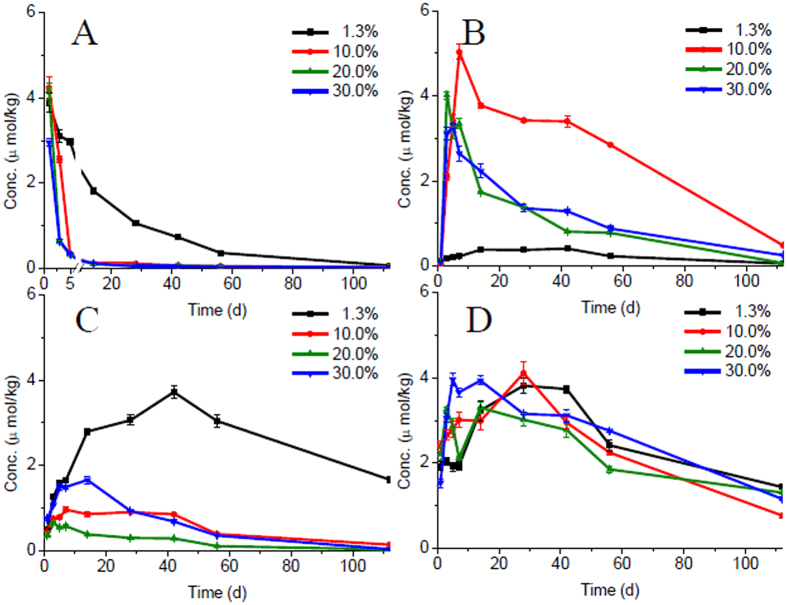
The effect of water content on the degradation of FE and its degradation products. (**A**) FE; (**B**) FA; (**C**) CDHB; (**D**) EHPP and HPPA.

Previous study has suggested that FE to FA was mainly attributed to hydrolysis^4^,^8^. In this work, the effects of water content on the degradation and enantioselectivity of FE and the degradation products were investigated. An incubation experiment involving four levels of water contents (air-dried (1.3%), 10%, 20% and 30%) under sterilized conditions was conducted for a 120-day period in order to summarize the influence of water content. Water content affected FE degradation in the sediment from reservoir significantly, and the degradation was much faster in the sediment with higher water content because of hydrolysis (Fig. 5A). A very small amount of FA was generated in the air-dried sediment which was consistent with the degradation of FE, and the further degradation was also slow (Fig. 5B). FA concentration was much higher in the sediment with water content of more than 10% and it was found the further degradation was faster with higher water content. The amount of CDHB in molality from the degradation of FE or FA should be equal to the amount of the sum of EHPP and HPPA theoretically because of the cleavage of the ether linkage of FE and FA. So the concentration-time curves were made from molality verse time, and the sum of EHPP and HPPA versus time was generated for comparison. The amount of CDHB was much higher in the air-dried sediment, maybe because the further degradation was slower in the sediment with low water content (Fig. 5C). The degradation products HPPA and EHPP seemed not relevant to water content (Fig. 5D) suggesting the formation and further degradation was not affected by water very much. The CDHB was lower than the sum of EHPP and HPPA with higher water content due to the effect of water content on the degradation of CDHB. The concentration-time curves in Fig. 5C,D were similar only in the air-dried sediment. The degradation products FA, CDHB, HPPA and EHPP could be detected after 120 days, in which EHPP were more persistent. The EF values of FE, FA, EHPP and HPPA remained unchanged from the initial value during the incubation period. The results showed the high water content in the sediment could accelerate the degradation of FE and degradation products without affecting the enantioselectivity under sterilized conditions. In a real environment, the slower degradation and more residues is likely to appear in water-starved reservoirs or during the dry season.

## Conclusions

The environmental fate of fenoxaprop-ethyl in water, sediment and water-sediment microcosm from reservoir was studied. Fenoxaprop-ethyl degraded rapidly with time in water, sediment and water-sediment microcosms, leading to the fast formation of the primary degradation product fenoxaprop. The degradation of fenoxaprop-ethyl, fenoxaprop, ethyl-2-(4-hydroxyphenoxy)propanoate and 2-(4-hydroxyphenoxy)propanoic acid were enantioselective and the enantioselectivity was attributed to the microorganisms in water and sediment. Fenoxaprop-ethyl tended to distribute in the sediment because of water solubility. The degradation products FA, CDHB, HPPA and EHPP were quite persistent in the environment. The impact of water content showed the high water content in the sediment could accelerate the degradation of FE and the degradation products. Further researches should be conducted to evaluate the toxicity of the degradation products.

## Materials and Methods

### Chemicals and materials

Racemic fenoxaprop-ethyl (rac-FE, 98.0%) with EF values 0.491 plus or minus 0.005 and (R)-(+)-fenoxaprop-ethyl (R-FE, 98.0%) were kindly supplied by Institute for Control of Agrichemicals, Ministry of Agriculture of China. Racemic fenoxaprop (rac-FA, 98.0%), (R)-(+)-fenoxaprop (R-FA, 98.0%), racemic ethyl-2-(4-hydroxyphenoxy)propanoate (rac-EHPP, 98.0%), (R)-(+)-ethyl-2-(4-hydroxyphenoxy)propanoate (R-EHPP, 98.0%), rac-2-(4-hydroxyphenoxy)propanoic acid (rac-HPPA, 98.0%), (R)-(+) -2-(4-hydroxyphenoxy)propanoic acid (R-HPPA, 98.0%) and 6-chloro-2,3-dihydrobenzoxazol-2-one (CDHB, 98.0%) were synthesized and characterized according to the reported procedure^15^,^16^,^17^. All other chemicals and solvents were purchased from commercial sources and were of analytical grade. FE stock solution of 1000 μg/mL was prepared by dissolving FE in analytical grade acetone purchased from Beijing Chemical Reagent Company Limited.

Water and sediment were sampled at Shangzhuang reservoir in Beijing, China. Sediment was air-dried, homogenized and sieved to below 2 mm. All experiments were carried out with the same batch of water and sediment. Water Quality was shown below: pH, 7.5; total alkalinity, 260 mg/L; suspended solids, 42 mg/L; total dissolved solids, 559 mg/L. Properties of the sediment shown below: pH, 8.3; clay, 10.0%; silt, 12.0%; sand, 78.0%; organic matter, 6.75 g/kg.

### Enantioselective and quantitative HPLC-MS/MS analysis

A method was developed for the determination of FE, FA, EHPP, HPPA enantiomers and CDHB based on high performance liquid chromatography HPLC-MS/MS (Thermo Scientific, TSQ Quantum Access Max). The chiralpak IC chiral column (250 × 4.6 mm, Daicel Chemical Industries) was used for analysis of FE, FA, EHPP, and CDHB while Lux Cellulose-3 chiral column (150 × 2.0 mm, Phenomenex) was used for separation of HPPA. The chromatographic conditions were listed in Table S2. Isocratic conditions were used and that each of these analytes was separated under the merthanol/water/formic acid mobile phase conditions in order to achieve separation of R-enantiomer and S-enantiomer. Three different mobile phase conditions were used for the analysis when chiralpak IC column was used. CDHB and FA were analyzed in the same run. The column temperature was 20 °C with injection volume of 5 *μ*L. Figure S4 showed the chromatograms of FE and its degradation products, and the elution order and identities were established by comparison with the retention time and fragmentation of the enantiopure standards of FE, FA, EHPP and HPPA and the standard of CDHB. The quantitative analysis was based on the response from the quantitative SRM. Mass spectral information about FE and its degradation products was listed in Table S2.

### Water, sediment, and water-sediment microcosm incubation

The incubation was carried on in natural water, sediment and a mixture of both from Shangzhuang reservoir. Water-sediment microcosm was constituted by 50 g of sediment (20% water content) and 250 mL of water in a 500-mL beaker, which was pre-incubated at 25 °C for 15 days before application of FE. The surface area of the sediment exposed to water was 56.7 cm^2^. Two contamination ways were applied (1) water contamination (1.38 μmol/kg): 125 *μ*L of rac-FE acetone solution (1000 μg/mL) was dropwise fortified in the water using a microsyringe; (2) sediment contamination (6.91 μmol/kg): separate the water and sediment in the microcosm first, add 125 *μ*L of rac-FE acetone solution (1000 μg/mL) to the sediment and mix thoroughly with a spatula, then transfer the water back as gently as possible without disturbing the sediment. The degradations in only sediment or water were also performed as control groups. Two hundred and fifty grams of water was fortified with rac-FE at 1.38 μmol/kg and 50 g of sediment (20% water content) was fortified with rac-FE at 6.91 μmol/kg. Sterilization treatments were also conducted to investigate the impact of microorganisms by autoclaving water and sediment samples twice at 120 °C for 60 min in 24 h intervals to eliminate microbial activity. In order to study the impact of water content on the degradation of FE in sediment, the water content from 1.3% to 30% was adjusted by adding water from Shangzhuang reservoir to air-dried sediment (1.3%). The air dried sediment water content (1.3%) was determined by weighing fresh soil samples, oven-drying at 105 °C for 4 days, and reweighing them to account for the loss in soil moisture^18^.

Triplicates were conducted and all of the samples were incubated in an incubator at 25 °C under a light/dark regime of 12:12. The samples were collected at different time intervals (day 0.25, 0.5, 1, 1.5, 2, 2.5, 3, 5, 7, 10, 14, 21, 28, 42, 56 and 84 after treatment) and immediately transferred into a freezer (−20 °C) to stop further degradation. Blank determinations of the water and sediment prior to fortification revealed the concentrations of FE and its degradation products in blanks were below limits of quantification (LOQs).

### Extraction of sediment and water

Samples were thawed at room temperature. For sediment, 10 g was placed into a 50-mL polypropylene centrifuge tube. After adding 20 mL of ethyl acetate and 100 *μ*L of 1.0 × 10^6^ *μ*mol/L hydrochloric acid, the tube was stirred for 10 min on a vortex mixer, ultrasonically extracted for 30 min, and then centrifuged at 3500 rpm for 3 mins. The same extraction step was repeated, and the solvent was filtered through 5 g of anhydrous sodium sulfate for dehydration. The combined extract was evaporated to near dryness in a vacuum rotary evaporator at 35 °C and reconstituted in 0.5 mL of methanol, which was filtered through a 0.22 μm film. For water, it was directly determined after filtration without extraction. An aliquot of 5 *μ*L was injected into HPLC-MS/MS.

The method was validated by linearity, recovery, limit of quantification (LOQ) and RSD. Good linearity was obtained over the concentration range of 0.06-1.5 μmol/kg in water and 0.09–4.5 μmol/kg in sediment for rac-FE, rac-FA, rac-HPPA, rac-EHPP and CDHB. Correlation coefficients (R) of linear regression equations were above 0.99. Recoveries were determined by blank sediment and water samples fortified with rac-FE, rac-FA, rac-HPPA, rac-EHPP, and CDHB at 0.1, 0.5 and 1.5 μmol/kg, which were above 80% based on an acceptable RSD less than 20%. The limit of quantification (LOQ) based on S/N = 10 for rac-FE, rac-FA, rac-EHPP, rac-HPPA and CDHB in water and sediment was listed in Table S2.

## Additional Information

**How to cite this article**: Jing, X. *et al*. Environmental Fate of Chiral Herbicide Fenoxaprop-ethyl in Water-Sediment Microcosms. *Sci. Rep.* **6**, 26797; doi: 10.1038/srep26797 (2016).

## Supplementary Material

Supplementary Information

## Figures and Tables

**Figure 1:**
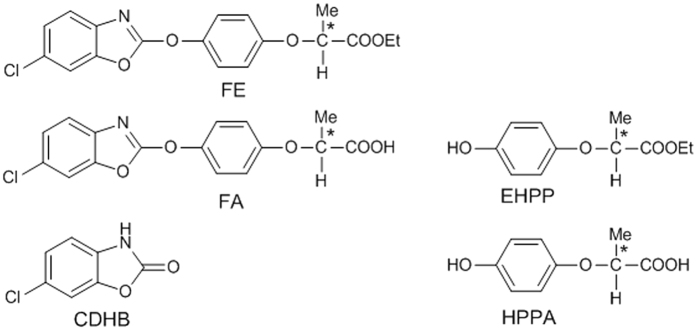
Chemical structures of fenoxaprop-ethyl (FE) and the four degradation products. *Indicates chiral center.

**Table 1:** Half-lives (hour) of FE and its degradation products in water and sediment.

	R-FE	S-FE	R-FA	S-FA	R-EHPP	S-EHPP	R-HPPA	S-HPPA	CDHB
Water	<0.25	<0.25	12.4	8.2	28.9	11.8	15.1	9.1	96.3
Sterilized Water	1.3	1.3	33.0	33.0	82.5	82.5	15.9	15.9	101.9
Sediment	0.7	0.4	9.4	6.4	63.0	33.0	3.4	3.2	17.8
Sterilized Sediment	1.4	1.4	19.8	19.8	67.3	67.3	16.6	16.6	22.2

Note: The degradation followed the first-order equation (C = C_0_e^−kt^) with high correlation coefficient. The half-life was estimated by the equation: t1/2 = In 2/k.
