# Supplementary material for: Environmental Fate of Chiral Herbicide Fenoxaprop-ethyl in Water-Sediment Microcosms
Source: Sci Rep. 2016 May 26;6:26797. doi: 10.1038/srep26797 (PMC4880935; doi:10.1038/srep26797)
Supplement: Supplementary Information [file srep26797-s1.doc]

Environmental Fate of Chiral Herbicide Fenoxaprop-ethyl in Water-Sediment Microcosms

Xu Jing, Guojun Yao, Donghui Liu, Mingke Liu, Peng Wang*, Zhiqiang Zhou*

Beijing Advanced Innovation Center for Food Nutrition and Human Health.

Department of Applied Chemistry, China Agricultural University, Beijing, 100193, PR China

*Co-corresponding author:

Peng Wang, Department of Applied Chemistry, China Agricultural University, No.2 Yuanmingyuan West Road, Beijing 100193, P.R. China; Tel: +8610-62731294; Fax: +8610-62732937; E-mail: [wangpeng@cau.edu.cn](mailto:wangpeng@cau.edu.cn)

Zhiqiang Zhou, Department of Applied Chemistry, China Agricultural University, No.2 Yuanmingyuan West Road, Beijing 100193, P.R. China; Tel: +8610-62733547; Fax: +8610-62733547; E-mail: zqzhou@cau.edu.cn

Table S1 General information of FE and FA

| Compounds | Solubility in water at 20°C  (mg/L) | log *K*ow | Vapour pressure  at 25°C (mPa) | Bulk density  (g/mL) | Henry's law constant at 20°C | GUS leaching potential index |
| --- | --- | --- | --- | --- | --- | --- |
| FE | 0.9 | 4.28 | 1.87 × 10^-04^ | 1.3 | 3.20 × 10^-07^ | 0.02 |
| FA | 61000 | 1.04 | 0.18 | 1.5 | 4.04 × 10^-10^ | 1.55 |

Table S2 HPLC-MS/MS conditions of FE and its degradation products

| Compounds | Chiral column | Methanol/water/ formic acid (v/v/v) | Flow rate (𝜇L/min) | Retention time (min) | | Precursor ion  (m/𝑧) | Product ions  (m/𝑧) | Collision energy (eV) | Spray  Voltage  (V) | LOQ  (water)  μmol/kg | LOQ  (sediment)  μmol/kg |
| --- | --- | --- | --- | --- | --- | --- | --- | --- | --- | --- | --- |
|  |  |  |  | R | S |  |  |  |  |  |  |
| FE | chiralpak IC | 85: 15: 0.1 | 500 | 24.1 | 25.7 | 362.1 | 288.1 | 17 | 3500 | 0.000164 | 0.000045 |
|  |  |  |  |  |  |  | 244.0 | 23 |  |  |  |
| FA | chiralpak IC | 75: 25: 0.1 | 500 | 17.5 | 19.1 | 332.0 | 260.0 | 16 | 3000 | 0.000249 | 0.000095 |
|  |  |  |  |  |  |  | 152.0 | 25 |  |  |  |
| EHPP | chiralpak IC | 50: 50: 0.1 | 500 | 25.1 | 27.1 | 211.1 | 137.1 | 10 | 3500 | 0.0144 | 0.0021 |
|  |  |  |  |  |  |  | 81.2 | 21 |  |  |  |
| HPPA | Lux Cellulose-3 | 40: 60: 0.1 | 200 | 13.5 | 15.6 | 181.0 | 109.1 | 20 | 3000 | 0.000598 | 0.000145 |
|  |  |  |  |  |  |  | 108.1 | 34 |  |  |  |
| CDHB | chiralpak IC | 75: 25: 0.1 | 500 | 11.5 |  | 168.0 | 132.1 | 20 | 3000 | 0.000476 | 0.000170 |
|  |  |  |  |  |  |  | 76.2 | 25 |  |  |  |


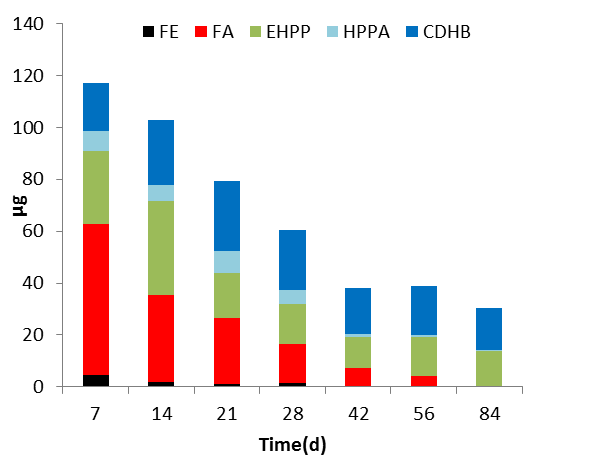

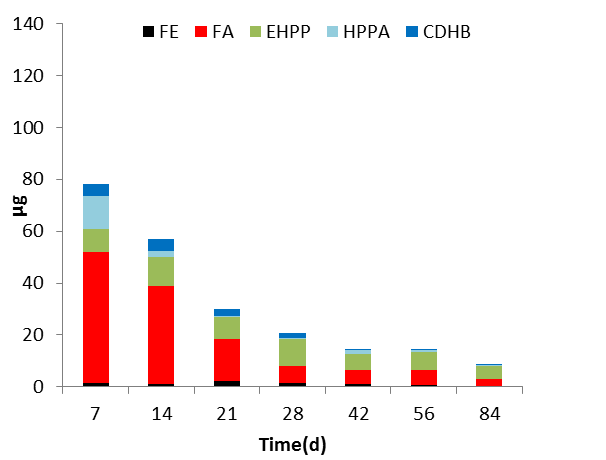

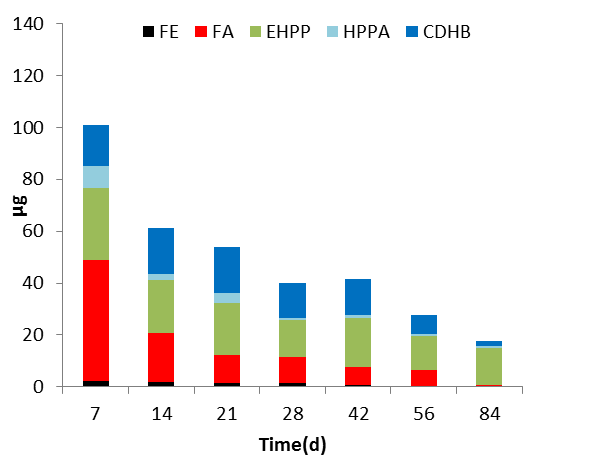

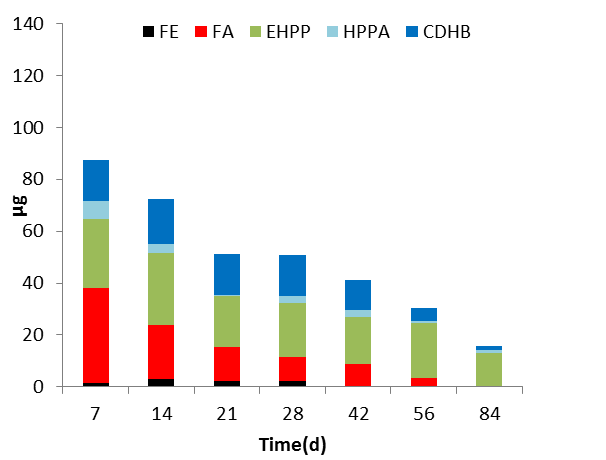


A B

C D

Figure S1. Material balance-time curves in (A) water; (B) sediment; (C) W-C microcosm ; (D) S-C microcosm.


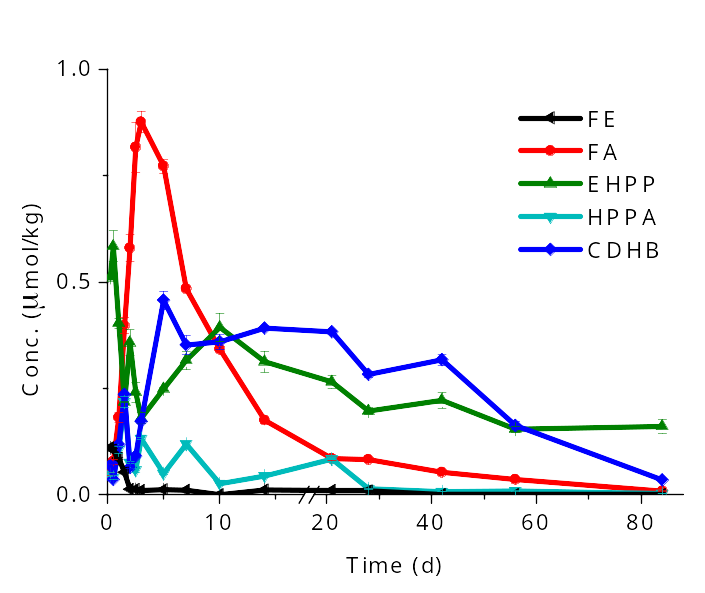

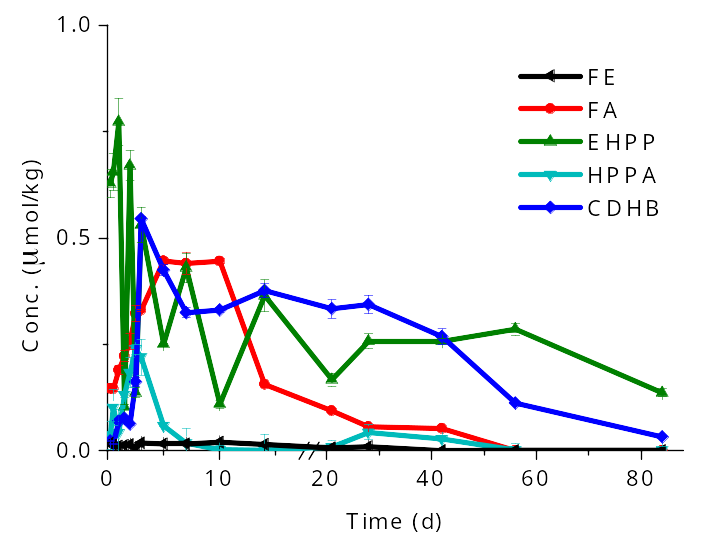

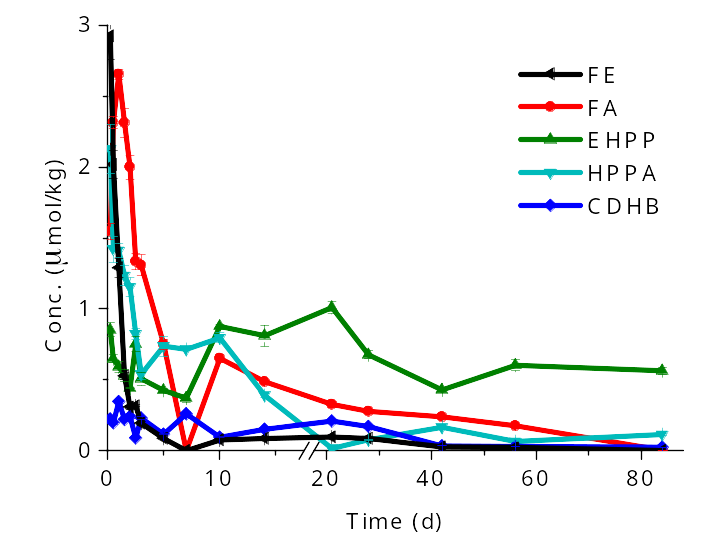

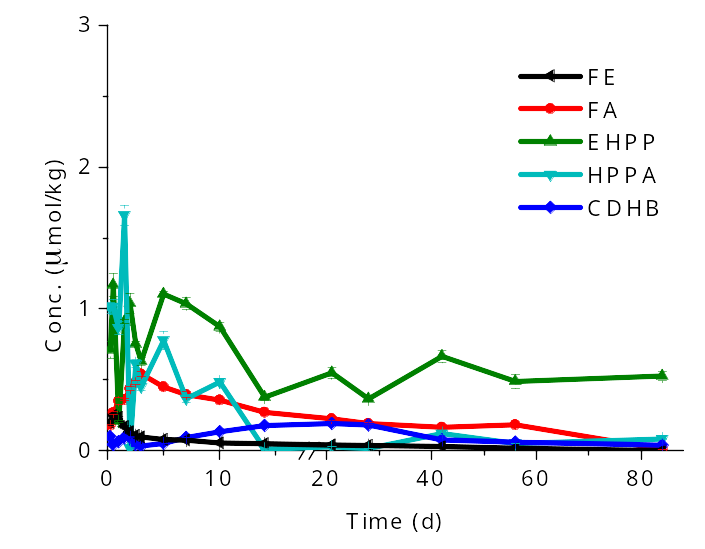


A B

C D

Figure S2. Concentration-time curves of FE and its degradation products in (A) water of W-C microcosm ; (B) water of S-C microcosm ; (C) sediment of S-C microcosm ; (D) sediment of W-C microcosm.


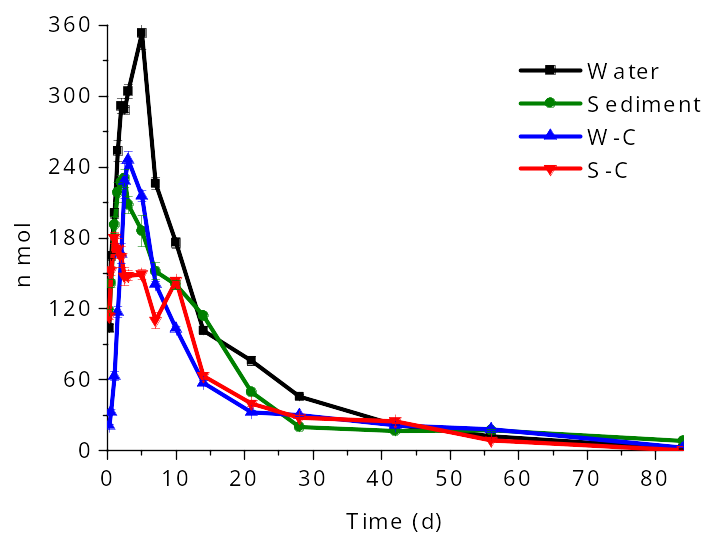

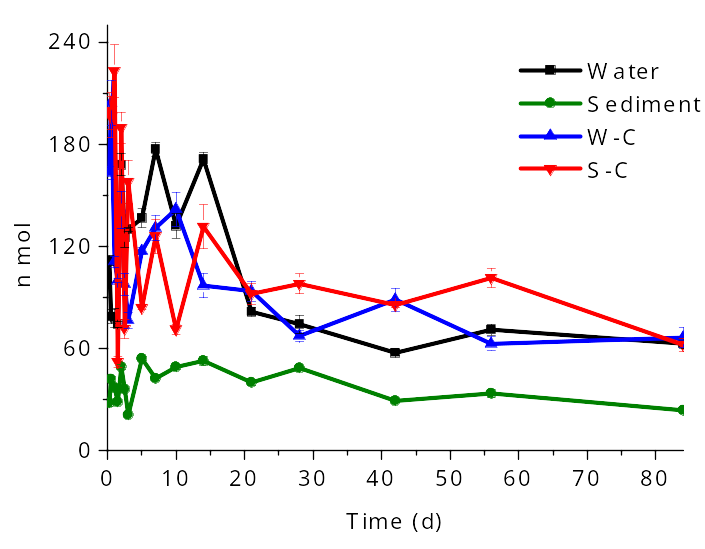

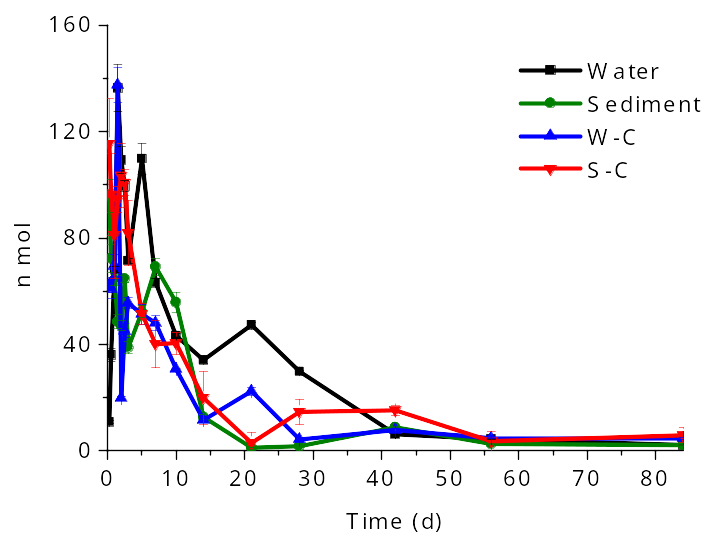

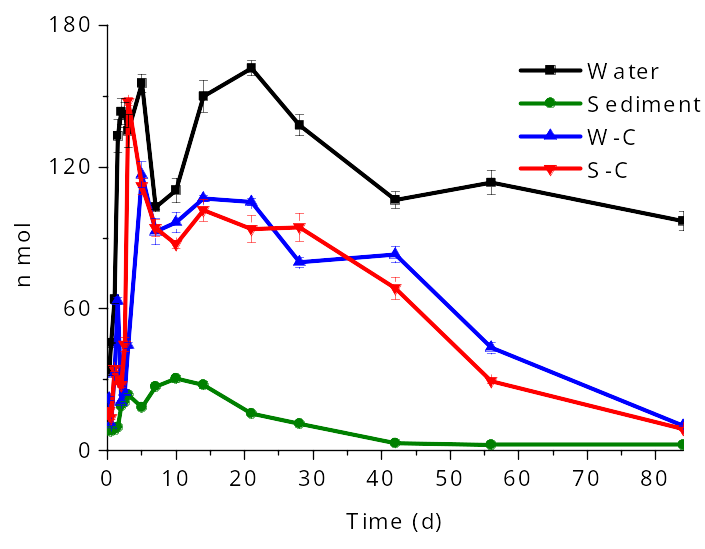


A B

C D

Figure S3. Amount-time curves of FE degradation products in water, sediment, W-C microcosm and S-C microcosm. (A) FA; (B) EHPP; (C) HPPA; (D) CDHB.


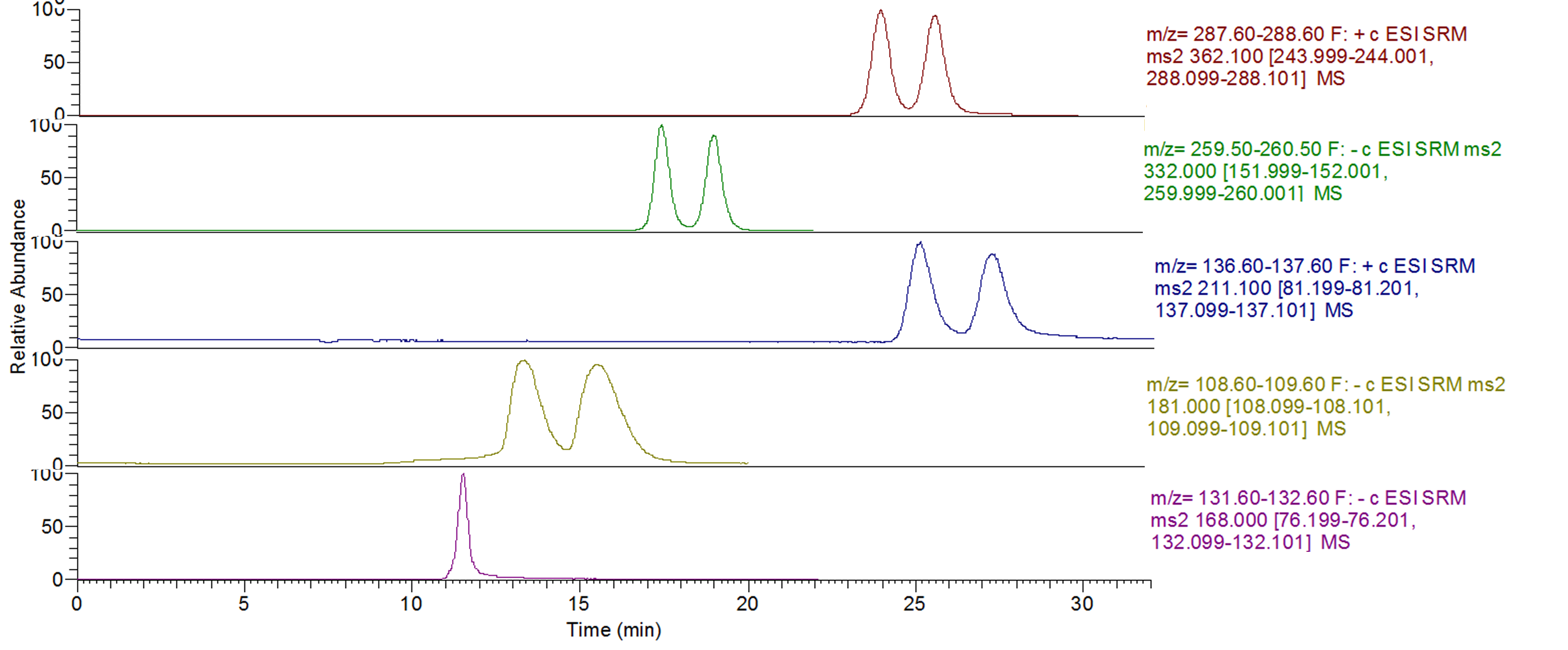


(A) FE

FA

EHPP

HPPA

CDHB


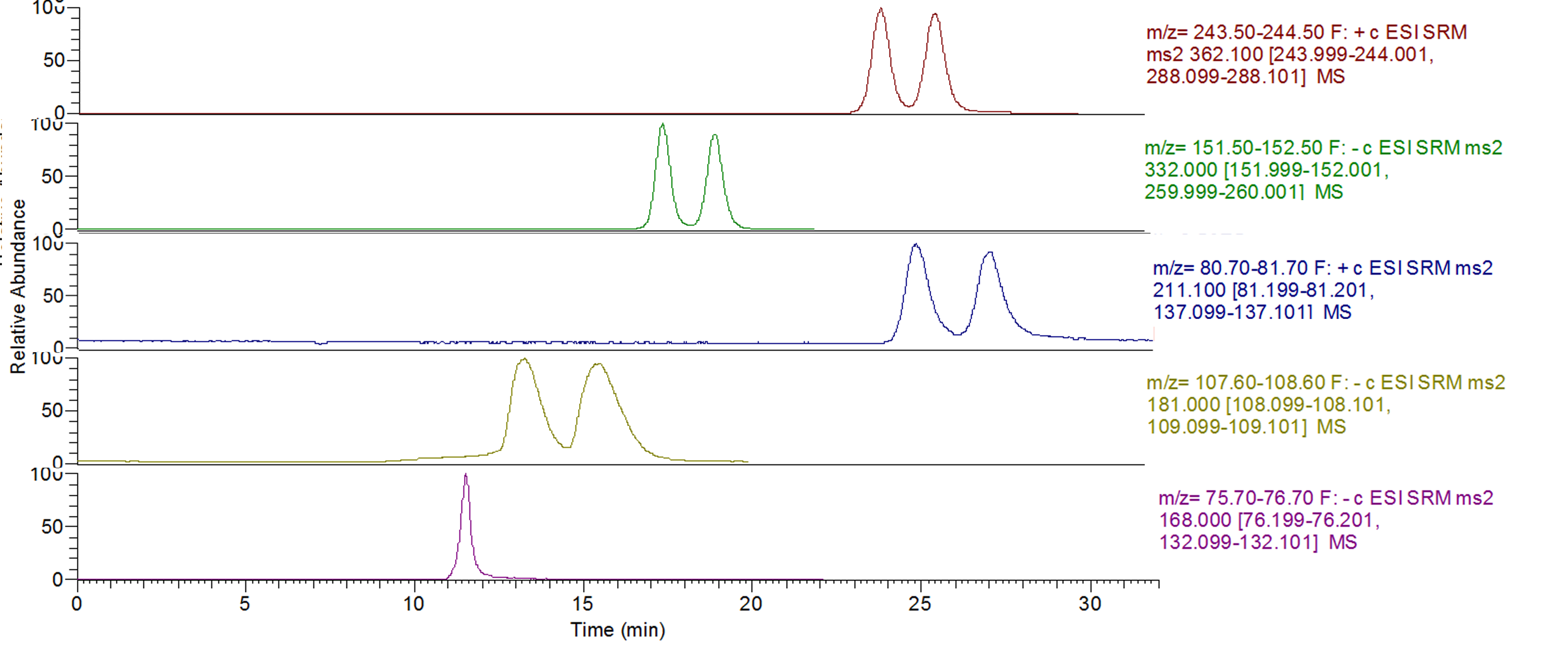


(B) FE

FA

EHPP

HPPA

CDHB

Figure S4. HPLC/MS-MS chromatograms of FE and its degradation products (10 mg/L methanol solution) (A) quantitative SRM; (B) qualitative SRM
